# The Commissioners in Lunacy and the Poor-Law Board

**Published:** 1860-01-01

**Authors:** 


### Art. VIII.—PAUPER LUNATICS.

I. The Commissioners in Lunacy and the Poor-Law Board.
II. Statistics.

### I.

In April last a Supplement to the Twelfth Report of the Commissioners in Lunacy was published, containing an account of the condition, character, and treatment of lunatics in workhouses. The matter of this supplementary report appeared to us at the time so important that we devoted an article to the question of Pauper Lunacy.* From the statistics accessible to us we came to the conclusion that there was a steady and progressive increase in the amount of *pauperism* from lunacy in the country; that the explanations advanced to show that this was more apparent than real were not satisfactory; and, on the authority of the Lunacy Commissioners, we ventured to express the opinion that it seemed as if our workhouses, in reference to lunatics, were "little better than *hot-beds* in which pauper lunacy was fostered and matured." The Lunacy Commissioners had, indeed, asserted that the detention of lunatics in these institutions was "one of the most fertile causes of the increase of lunatic paupers throughout the country. It is this that mainly tends to fill our county asylums with hopeless chronic cases, and is most directly responsible for the heavy and permanent burdens upon parish rates."† Moreover, in his evidence before the Select Committee on Lunatics, Session 1858-59, Lord Shaftesbury, the Chairman of the Lunacy Board, had stated the condition of lunatics in workhouses in stronger terms even than those used in the Supplementary Report of the Commissioners.

The very grave charges advanced by the Commissioners have not been suffered to pass unnoticed by the Poor-Law Board; and Mr. Andrew Doyle, one of the Inspectors of the Board, gave evidence before the last Select Committee on Lunatics, with an intention to show that the Lunacy Commissioners had been guilty of great exaggeration in what they had reported concerning workhouses. As we had conceived that, from the evidence adduced in the text and appendix to the Commissioners' Report, we were warranted in assuming the correctness of their conclusions, it is but just that we should put our readers in possession of the principal objections made by Mr. Doyle.

This gentleman characterizes the statements of Lord Shaftesbury and the Commissioners' Report in the following terms:—

"I have to observe of the evidence of Lord Shaftesbury, as of the statements published by the Commissioners, that they are general allegations, almost impossible to lay hold of, but that whenever I have been able to put my finger upon particular statements, I have found nine out of ten, in some important respect or other, at variance with the facts as I have found them upon investigation.

"Do you confine your contradiction to your own district only, or do you

* See Vol. xii. p. 337.
† Supplementary Report, p. 34.

apply it to the rest of the kingdom generally?—I apply it emphatically to the whole of the kingdom, upon evidence which I am prepared to give before the Committee. I shall show that the cases selected by the Commissioners, as illustrations of the bad condition of workhouses, are the only cases that have occurred, and that their complaints are, in nine out of ten cases, inconsistent with evidence in the office of the Commissioners in Lunacy. That I am prepared to prove."*

Mr. Doyle, however, makes an important distinction between the Special Reports of the Commissioners on different workhouses and their General Report. He says:—

"As I have stated before, I have not found, so far as I can judge, one word of exaggeration from the beginning to the end of the Reports of the Commissioners. Accidental mistakes I have found. *But I have found exaggeration, misstatements, and incorrect quotations of the Reports of the Commissioners in this Supplemental Report.* Of the separate Reports of the Commissioners, as to their impartiality and general fairness, I cannot speak too highly. It is impossible that any document can contrast in those qualities more remarkably with them than the Supplemental Report which professes to be founded upon them."—(Qy. 1983.)

In support of these assertions, Mr. Doyle instanced, first, a statement made respecting the Blackburn workhouse, at p. 23 of the Supplemental Report. It is there said that, "at an inclement season of the year, there was not more than one blanket on any bed, and on many there were none whatever." In the special reports on the Blackburn workhouse contained in the appendix to the General Report, it is stated, under the date of 29th October, 1859, simply, that "only one blanket is allowed to each patient in the coldest weather;" and under date of 31st July, 1858, that the beds were clean, "but there is no blanket either above or below the patients." Mr. Doyle remarks that when the last statement was referred to the guardians, their answer was, "that there were no blankets upon the beds, in consequence of the heat of the weather, the paupers having requested that they might be taken off." This was given as one illustration of inaccuracy and misstatement, and may be fairly granted.

Again, the Commissioners recorded several examples of the dangers arising from discharging imbeciles or idiots from workhouses without medical sanction. In the Newark workhouse there had been found, "among other instances," two females, "who, although classed as of weak mind, were in the habit of discharging themselves, and after a short absence returning in the family way." Upon this statement, Mr. Doyle remarks—"That is perfectly true; the guardians assumed that they had no right to detain them." In the Walsall workhouse an idiotic female was found who had had four illegitimate children. It would appear, however, that the youngest was six years old before the woman was admitted to the workhouse. This, therefore, is not a legitimate illustration. In the Monmouth workhouse two imbecile paupers were met with, "each of whom had had three illegitimate children, and one of whom was again pregnant." To this Mr. Doyle answers, that "*they were not certified as idiots, and the guardians could*

* Report of Select Committee on Lunatics, Session 1859. Queries 1972—1974.

*not detain them.*" This is simply a quibble. In the Tamworth workhouse there were two idiotic women who had each had a child, but it would seem that they first came into the workhouse pregnant with those children. The last case referred to by Mr. Doyle occurred at the Mortley workhouse, in Worcestershire. "A female who had for some time been classed as of weak mind, was struck off the list in 1856, and was allowed to leave the house for the purpose of saving expense to the parish by earning something at hop-picking. This woman had *previously had two illegitimate children by paupers in the house*, one of whom had died; the other (a girl about ten years of age) she took with her, on quitting the house, to her mother's home. When there, she and her daughter slept in the same room with her father-in-law and her mother, and in the same bed with two of her brothers. The result of this indecency was, that she returned to the workhouse in the family way, and was delivered of a child, the father of which she distinctly stated to be one of her brothers, but which she was unable to specify. This woman, though able to perform some useful work, *was decidedly of weak mind*, and there can be no doubt that, under the circumstances, the guardians were justified in detaining her in the workhouse, and that they ought not to have sanctioned her quitting it." Mr. Doyle's explanation of this is, that the woman was not classed as an idiot in 1856, she having been struck off the list of idiots, by the medical officer in October, 1855, and that "the statement that the guardians had the power to detain her was perfectly preposterous, even if their medical officer had not certified that she was not an idiot."—(Qy. 1997.) This may explain the mode in which freedom of action was given to an erotic imbecile, but it is no justification of that freedom. For the credit of humanity we will admit that the guardians did not allow the woman to leave the workhouse in 1856 "for the sake of saving a few shillings;" but can we justify them for not having taken the necessary steps to remove her to an asylum if she could not be legally detained in a workhouse? Could any argument show more emphatically than this last story and the exculpation offered for it, the unfitness of workhouses for the confinement of lunatics or imbeciles. This woman had had two children to paupers in the workhouse (a curious comment on the propriety of keeping persons of weak mind in these institutions), and although manifestly an erotic imbecile, as the remarks of the visiting Lunacy Commissioner and the history of the case show, she is allowed to leave the house uncontrolled, under the cover of a legal quibble—for Mr. Doyle's objection amounts to nothing more than that. We admit the inaccuracy of the illustrations from Walsall and Tamworth; but Mr. Doyle's explanations have clenched the truthfulness of the remainder.

Mr. Doyle next makes a trifling objection to a remark concerning the Blackburn workhouse, and he then proceeded to the question of restraint, and said:—

"I find it stated by the Commissioners that mechanical control 'is habitually employed to restrain the idle and mischievous propensities of patients.' I have gone through the whole of the Reports of the Commissioners in Lunacy (for two years) that were accessible to me, nearly 600 out of the whole 650, and I find the number in which they complain of restraint being imposed *is only*

*nineteen from the whole of the workhouses to which they refer.* . . With the exception of, I think, three, every one of those cases of restraint being, in my opinion, open to satisfactory explanation."—(Queries, 2015, 2017.)

The Commissioners had asserted that the law respecting the removal of pauper lunatics to asylums was constantly evaded; Mr. Doyle states that of the total number of 7000 lunatics found by the Commissioners in workhouses during the period referred to in their report, "they recommended the removal of only 153."—(Qy. 2018.)

These objections are certainly of considerable importance, and would incline us to modify our views in several respects as to the *extent* to which the evils animadverted upon by the Lunacy Commissioners exist.

Mr. Doyle is less happy in several other objections, but we need only mention one of these. He remarks, in reference to certain observations in the Supplementary Report which "would lead any man to state that the poor in workhouses are 'stinted or starved;'" that "nobody who is acquainted with the practice of the Poor Law Board can appreciate the pains they take to ascertain that every dietary table which is issued contains an average quantity of nutritious food. In point of fact, every dietary table of every union in England is submitted to the Poor Law Board; it is analysed scientifically by them before it is issued, and upon that analysis they frequently make suggestions for improvement; and no dietary table can be issued in any workhouse without having the seal of the Poor Law Board, after having undergone that examination, affixed to it."—(Qy. 2056.)

Mr. Doyle had surely forgotten the examples of *minimum* workhouse diets recorded in the Supplementary Report when he made this statement. At the Hailsham workhouse the lunatics have but one spare meat dinner during the week, four ounces of meat only being allowed to the men, and two and a half ounces to the women; while bread and cheese, without beer, constitute the dinners on four other days, and pudding on the remaining two. In the Amesbury workhouse the inmates are restricted to bacon twice a week (four ounces for men, and three ounces for women) pudding twice, and a very weak soup twice. "In many other workhouses," the Lunacy Commissioners assert, "meat is only given to the insane once a week, and even then in very small and insufficient quantity."* Mr. Doyle seems also to have forgotten that, shortly before he made his statement respecting workhouse dietaries, the following passage had been read to him from a report made by three local magistrates, concerning the treatment of lunatics in the workhouse at Dursley.

"We saw and ascertained that about one quarter of the dinner given to the females in the idiot ward consisted of boiled sweed turnips, an article grown for the consumption of cattle and sheep, but not of mankind; that it is used for the inmates generally, as well as for the three or four females of this class, may be inferred from the strong smell arising from its being boiled pervading the house.

"Purnell B. Purnell."

* Supplementary Report, p. 18.

Mr. Doyle and the Commissioners of Lunacy regard the subject of the care of lunatics in workhouses from very different points of view. The latter use the term lunatic in its widest signification; the former would restrict its meaning very considerably, hence it is that he says:—"In nine out of ten unions I find no lunatics at all, or only two or three harmless people out on the land working, and if they are women, in the body of the house, doing household work; in fact, they are servants of the establishment, and not lunatics or idiots in the sense in which any man of common sense would call them so; they may be brought, by the very large definition given by the Commissioners of Lunacy to the medical officers, within the scope or the meaning of lunatics and idiots, but lunatics and idiots, properly speaking, they are not."—(Query 2054.) Unfortunately, Mr. Doyle had a few moments before spoken thus of his knowledge of lunacy:—"I have no knowledge whatever, and I have no pretension to give an opinion upon the question with respect to the treatment of lunacy or lunatics.—Or with regard to the characteristics of insanity?—Certainly not."—(Queries 2035, 2036.) Common sense, therefore, as used by Mr. Doyle in the above expression of opinion, must mean entire ignorance. The Commissioners conceive that workhouses cannot, under any circumstances, become a satisfactory place for the care or treatment of lunatics or idiots. Mr. Doyle believes that these institutions are well fitted for the care of idiots, imbeciles, and chronic cases of insanity; but by the announcement of his ignorance both of the characteristics and treatment of the insane he has cut the ground from under his feet entirely. The Commissioners knowing what insanity is, and what is required even for the ordinary care of the mildest or the most confirmed case, and seeing how impracticable it is to mould the rules necessary for the government of a workhouse, or the accommodation requisite for paupers, into a form adapted for lunatics, idiots, or imbeciles, have concluded the worst from the facts ascertained during their inquiry. They have painted their picture, therefore, in gloomy colours. Mr. Doyle has evidently never before given much attention to the lunatic inhabitants of workhouses; he regards the workhouse as the proper place for the pauper; he can see no reason from want of previous attention to the subject, why the quiet lunatic, idiot, or imbecile should not be treated simply as a pauper; he has admitted his ignorance of the very points which would alone have enabled him to judge rightly of the general question, but strong in his convictions, he has opposed a vigorous, if not very coherent, defence against the assertions and conclusions of the Commissioners. He has said amply sufficient to show (if any one, indeed, needed that showing) that Lord Shaftesbury was not justified in asserting that the idiots in our workhouses were exposed to the "very greatest cruelty," and that "we are now returning, in these workhouses, to the system of things that prevailed in 1828, there being no means of classifying these persons; a large proportion of these were then chained down, and kept in the most horrible state of filth and suffering." He has said sufficient also to convince us that the impression we had derived from the Supplementary Report respecting the general treatment of lunatics in workhouses

Bourne fo is a somewhat exaggerated one, and so far wrong; but he has said nothing to convince us that the evils detailed in that Report are not peculiar to, and probably inseparable from, the rules regulating workhouse management, and that the opinions founded upon these evils are not, at the bottom, essentially correct. Mr. Doyle has painted his subject in the brightest colours, but we cannot avoid thinking that he has helped to bring out in a clearer light the unfitness of workhouses either for the detention or treatment of lunatics.

With the exception then of modifying our notions somewhat as to the extent to which the grosser evils arising from the detention of lunatics in workhouses prevail, we see no need to alter the conclusions we arrived at in our article on "Pauper Lunacy."

